# The Preventive Power of the Mediterranean Diet Against Blue-Light-Induced Retinal Degeneration: Is the Secret in the Herbs and Spices?

**DOI:** 10.3390/cimb47060418

**Published:** 2025-06-04

**Authors:** Anja Harej Hrkać, Ana Pelčić, Tea Čaljkušić-Mance, Jasenka Mršić-Pelčić, Kristina Pilipović

**Affiliations:** 1 Department of Basic and Clinical Pharmacology and Toxicology, Faculty of Medicine, University of Rijeka, 51000 Rijeka, Croatia; aharej@medri.uniri.hr (A.H.H.); jasenka.mrsic.pelcic@medri.uniri.hr (J.M.-P.); 2 Faculty of Medicine, University of Rijeka, 51000 Rijeka, Croatia; apelcic1@student.uniri.hr; 3 Department of Ophthalmology, Faculty of Medicine, University of Rijeka, 51000 Rijeka, Croatia; tea.mance.caljkusic@medri.uniri.hr; 4 Clinic for Ophthalmology, Clinical Hospital Center Rijeka, 51000 Rijeka, Croatia

**Keywords:** diet, Mediterranean, blue light, macular degeneration, retinal degeneration

## Abstract

The Mediterranean diet, rich in plant-based foods, healthy fats, and herbs, has long been associated with a range of health benefits, including cardiovascular, neuroprotective, and anti-inflammatory effects. Recent studies suggest that certain components of this diet, particularly spices such as bay laurel, thyme, oregano, sage, and rosemary, may play a critical role in protecting the retina from oxidative damage, a key factor in blue-light-induced retinal degeneration. Blue light, emitted by digital screens and artificial lighting, has been implicated in the development of retinal conditions like age-related macular degeneration by inducing oxidative stress and inflammation. This review explores the potential of the herbs and spices commonly present in the Mediterranean diet to mitigate blue-light-induced retinal damage. These herbs are rich in polyphenols, flavonoids, essential oils, and terpenes, which offer antioxidant, anti-inflammatory, and antimicrobial properties, contributing to retinal health and reducing oxidative damage. By focusing on bioactive compounds such as eucalyptol (1,8-cineole), rosmarinic acid, carnosic acid, eugenol, and thymol, this article investigates how these herbs and spices might act as natural protectants against blue-light-induced stress and retinal degeneration. The findings highlight the promising role of these culinary staples in preventing retinal damage and offer insights into future dietary recommendations for eye health in an increasingly digital world.

## 1. Introduction

In recent nutrition research, the focus has shifted from examining the effects of individual nutrients or foods to exploring overall dietary patterns [1]. This change reflects the growing belief that a combination of foods and nutrients, through their synergistic interactions, plays a more significant role in influencing health outcomes than isolated nutrients alone.

The Mediterranean diet (MedDiet) is a term coined to identify a dietary pattern originating from the traditional cuisines of countries located around the Mediterranean Sea. It is most closely associated with the diet of Crete, much of Greece, and southern Italy from the early 1960s [2]. This diet, rich in whole plant foods, healthy fats, and fish, is recognized for its health benefits and is considered more than just a set of dietary guidelines, incorporating social, cultural, economic, and environmental aspects. In 2010, UNESCO declared the MedDiet an Intangible Cultural Heritage of Humanity [2,3]. In the initial document, four countries were mentioned—Greece, Italy, Morocco, and Spain—and the list was later expanded in 2013 by adding Croatia, Cyprus, and Portugal.

Key features of the traditional MedDiet include a high intake of plant foods; choosing fresh fruit as a typical dessert, with sweets containing sugars or honey being consumed rarely; a high intake of olive oil as the main source of fat (especially virgin and extra virgin); a moderate intake of dairy products (mainly in the form of cheese and yoghurt), with only up to four eggs consumed per week; a low to moderate intake of fish and poultry; a low intake of red meat; and moderate consumption of wine with meals [4].

Beyond the foundational foods of the MedDiet, it is important to note that Mediterranean cuisine is known for the use of predominantly locally sourced spices and aromatic herbs, as well as garlic and onion [5]. These are widely employed to enhance the palatability of plant-based dishes, which are central to the MedDiet. Rich in bioactive compounds (particularly flavonoids and polyphenols but also sulfur-containing substances, tannins, alkaloids, phenolic diterpenes, and vitamins), they may play a contributory role in the diet’s well-documented health benefits. Namely, plant- and spice-derived bioactive compounds have been shown to exert multiple beneficial effects, such as antioxidative and anti-inflammatory actions, the inhibition of tumor growth and carcinogenesis, and the modulation of metabolic parameters, including blood glucose and lipid profiles [4].

Digital lifestyles, characterized by the prolonged and frequent use of computers, smartphones, tablets, and other digital screens, have a significant impact on eye health. These effects can manifest as temporary discomfort and digital eye strain, also known as computer vision syndrome, a widespread multifactorial condition characterized by tired, dry, or burning eyes and blurred vision, but also as symptoms such as headaches and neck or shoulder pain [6,7]. Regarding their chronic effects, there is still no definite evidence on whether the excessive use of devices with digital screens causes permanent eye damage [8]. However, there are some indications that chronic exposure to digital devices may exacerbate the symptoms of underlying eye diseases [7,9,10]. Additionally, there are also concerns about whether screens’ blue light might be associated with eye damage [6,7,9].

The focus of this narrative review is exploring the protective potential of herbs and spices from the Mediterranean diet and their bioactive compounds against blue-light-induced retinal damage. By examining the interplay between modern lifestyle challenges, such as increased exposure to digital screens, and the traditional dietary practices of the Mediterranean region, we aim to highlight nutritional strategies that may contribute to preserving retinal health and preventing vision-related disorders.

## 2. The Mediterranean Diet: Key Components and Health Benefits

Designated by UNESCO as an Intangible Cultural Heritage of Humanity, the MedDiet is more than just a dietary pattern. It represents a holistic lifestyle deeply rooted in the culinary traditions, social practices, and cultural identities of the Mediterranean region, especially Greece and Southern Italy. As detailed in articles by Guasch-Ferré and Willett [4] and Sikalidis et al. [11], this way of eating emphasizes an abundance of plant-based foods. Vegetables, fruits, whole grains, legumes, nuts, and seeds form the cornerstone of the diet, providing significant amounts of fiber, vitamins, minerals, and antioxidants. Extra virgin olive oil, rich in healthy monounsaturated fats, is not merely an ingredient but a central element, used generously in cooking and as a flavorful dressing. Herbs and spices, such as basil, rosemary, oregano, thyme, cilantro, fennel, and mint, are preferred over salt, adding both distinctive flavors and a wealth of beneficial phytochemicals. The diet also includes moderate amounts of fish and poultry, with limited consumption of red meat and dairy products [4]. Red wine is often enjoyed in moderation with meals, rounding out the traditional Mediterranean dining experience [12].

The MedDiet is not completely uniform across the region. For example, while plant-based foods at the center and olive oil as the primary fat are constant elements, there are some variations in the uses of particular types of food. For example, in North Africa, couscous, vegetables, and legumes are important, unlike in Southern Europe, where pasta, polenta, rice, or potatoes with vegetables and legumes are heavily consumed. Also, in some cultures, alcoholic beverages are not included, mostly due to religious beliefs [4].

This dietary pattern, combined with regular physical activity and a socially engaging lifestyle, contributes to its well-documented health-promoting effects.

### 2.1. The Beneficial Effects of the Mediterranean Diet on Cardiovascular Health

Multiple large-scale epidemiological studies and randomized clinical trials, including the Seven Countries Studies, the Lyon Diet Heart Study, and the PREDIMED trial, have demonstrated the MedDiet’s efficacy in reducing the risk of cardiovascular disease or cardiovascular events by up to 30% in high-risk individuals [11]. Participants adhering to this diet showed a significant reduction in the incidence of myocardial infarction, stroke, and cardiovascular mortality.

It has been shown that the MedDiet improves plasma lipid profiles by increasing HDL cholesterol and lowering LDL cholesterol. Monounsaturated fats, especially from extra virgin oil and omega-3 fatty acids, reduce triglyceride levels, LDL oxidation, and lipid particle size, all factors that are crucial in preventing plaque formation in the arteries. Food rich in polyphenols (olive oil, red wine, vegetables, and fruits) inhibits the expression of pro-inflammatory genes [13], i.e., reduces the levels of inflammatory markers like C-reactive protein (CRP), interleukin-6 (IL-6), and tumor necrosis factor-alpha (TNF-α), and activates nuclear factor-kappa B (NF-κB) expression, a key regulator of inflammation in the endothelial cells and macrophages. High intake of potassium, magnesium, fiber (from fruits, vegetables, and legumes), vitamins C and E, carotenoids, and polyphenols inhibits platelet-activating factor (PAF) and improves antioxidant defense, the bioavailability of nitric oxide, and endothelial function, all crucial for maintaining vascular responsiveness and preventing hypertension and atherosclerosis. While omega-3 fatty acids and polyphenols reduce platelet aggregation, low-glycemic-index food like whole grains, legumes, and vegetables reduces postprandial glycemia and insulin secretion, thus lowering the risk of metabolic syndrome and diabetes as one of the major cardiovascular risk factors [11]. The synergistic effects of all the above—lipid-lowering, anti-inflammatory, antioxidant, and antihypertensive actions—contribute to a significant reduction in the progression of atherosclerotic disease and cardiovascular risk.

The PREDIMED (Prevención con Dieta Mediterránea) trial, published in 2013 by Estruch et al. in the New England Journal of Medicine, confirmed the significant cardiovascular impacts of the MedDiet [14]. This controlled trial randomized 7447 subjects aged 55–80 years old at high cardiovascular risk but with no prior cardiovascular disease. They were randomly assigned into one of three groups: the MedDiet with added extra virgin olive oil, the MedDiet with mixed nuts added to it, or a control group that was told to follow a low-fat diet. Over a median follow-up of 4.8 years, the subjects on the MedDiet experienced about a 30% reduction in their risk of major cardiovascular events (heart attack, stroke, or cardiovascular death) versus that in the control group. This research also documented improvements in their blood pressure, lipid profiles, and inflammatory markers. These findings strongly attest to the fact that the MedDiet, particularly when supplemented with healthy fats like olive oil or nuts, is highly effective for the prevention of cardiovascular disease in high-risk populations [15].

### 2.2. The Neuroprotective Effects of the Mediterranean Diet

Emerging research and supporting clinical evidence [16,17] indicate the MedDiet’s neuroprotective role in maintaining cognitive health and reducing the risk of cerebrovascular events or neurodegenerative disorders such as Alzheimer’s disease and Parkinson’s disease [18]. A high intake of polyphenols from fruits, vegetables, olive oil, and red wine, along with omega-3 fatty acids from fish, has been shown to reduce oxidative stress and chronic inflammation, both of which are implicated in neurodegenerative processes. These nutrients contribute to improved endothelial function and cerebral blood flow, thus reducing the risk of vascular-related cognitive impairments. It has also been shown that the MedDiet can reduce amyloid-beta accumulation and enhance neuronal signaling. Several studies have indicated that individuals who follow a Mediterranean-style diet perform better in cognitive tests and exhibit slower rates of brain atrophy and white matter degeneration [19]. This protective effect is thought to arise from the diet’s ability to modulate brain-derived neurotrophic factor (BDNF) and support synaptic plasticity, neurogenesis, and mitochondrial function, which are essential to the processes of learning and memory. Recently, it was described that the MedDiet, due to its high fiber content, fosters a healthy gut microbiome, which can influence brain health through the gut–brain axis.

A significant randomized controlled trial, the MedLey study, investigated the impact of the MedDiet on cognitive function among healthy older adults. The participants on the MedDiet showed significant improvements in several aspects of cognition, including memory, executive function, and processing speed, compared to those in the participants on a typical diet for six months. Such findings suggest that the MedDiet may enhance cognitive ability among the elderly [20].

A large-scale prospective cohort study utilizing data from the UK Biobank examined the relationship between MedDiet adherence and dementia risk. This study found that individuals with higher adherence to the MedDiet had a 23% lower risk of developing dementia, independent of genetic predisposition.

### 2.3. The Anti-Inflammatory Properties of the Nutrients Used in Mediterranean Cuisine

Chronic low-grade inflammation is implicated in the etiology of numerous chronic diseases, including cardiovascular disease, diabetes, cancer, and autoimmune conditions. The MedDiet’s rich content of anti-inflammatory compounds, including omega-3 fatty acids, polyphenols, flavonoids, and carotenoids, has been shown to suppress oxidative stress and key inflammatory pathways. Clinical evidence supporting the anti-inflammatory effects of the MedDiet [21] shows that regular intake of this dietary pattern is associated with decreased circulating levels of inflammatory biomarkers such as C-reactive protein (CRP) and pro-inflammatory cytokines such as IL-1β, IL-6, and TNF-α. The MedDiet also promotes the production of anti-inflammatory mediators like IL-10. The high fiber intake from whole plant foods also supports healthy gut microbiota, which play a crucial role in modulating systemic inflammation and immune responses. Namely, the fermentation of fiber by gut bacteria produces short-chain fatty acids like butyrate, which have anti-inflammatory properties and help maintain intestinal barrier integrity [22]. One of the possible mechanisms of the MedDiet’s anti-inflammatory actions is linked to an increase in adiponectin levels and a reduction in the levels of leptin, a hormone that has recently emerged as a key mediator between metabolic responses and low-grade chronic inflammation [23]. Through a reduction in the expression of adhesion molecules like ICAM-1 and VCAM-1, the MedDiet contributes to decreasing leukocyte adhesion and vascular inflammation [24,25].

Another study by Vaziri from 2024 emphasized the MedDiet’s potential as a powerful defense against Alzheimer’s disease [18]. The diet’s rich composition of antioxidants, healthy fats, and anti-inflammatory compounds contributes to its protective effects on cognitive function. Potential mechanisms might involve vascular factors, glucose/lipid metabolism, and anti-inflammatory effects.

## 3. The Eye-Related Benefits of the Mediterranean Diet

Recent evidence suggests that the MedDiet offers significant benefits for ocular health. This is particularly relevant given the increasing prevalence of vision-threatening conditions such as age-related macular degeneration (AMD) and diabetic retinopathy (DR), both of which are strongly influenced by oxidative stress, inflammation, and microvascular dysfunction, as described above [26].

### 3.1. Age-Related Macular Degeneration

AMD is the leading cause of blindness among older adults and is characterized by the progressive degeneration of the macula, the central portion of the retina. It is the leading cause of blindness in older adults, particularly in industrialized countries, and is projected to affect 288 million people globally by 2040, with Asia bearing the highest burden [27]. AMD is characterized by damage to the macula, the central part of the retina responsible for detailed vision. There are two main forms of AMD: dry (atrophic, non-neovascular) and wet (neovascular). Dry AMD is characterized by the accumulation of drusen and gradual atrophy of the retinal pigment epithelium (RPE) cells, while wet AMD involves abnormal blood vessel growth under the retina and more rapid vision loss. Both types involve degeneration of the RPE cells and photoreceptors, processes that are closely linked to oxidative stress and chronic inflammation.

Recent research indicates that individuals with higher adherence to the MedDiet are at a lower risk of developing AMD. Nutrients such as lutein, zeaxanthin, vitamin C, vitamin E, and zinc, abundant in leafy greens, citrus fruits, nuts, and seeds, play a key role in retinal protection [28].

Moreover, the anti-inflammatory and antioxidant properties of olive oil and polyphenol-rich foods help reduce the oxidative damage that contributes to retinal cell death. The beneficial fats from fish and nuts also support the structural integrity of the retinal membranes. Clinical studies, including those conducted in Mediterranean populations, have demonstrated slower progression of AMD among individuals who follow this diet [29,30].

### 3.2. Diabetic Retinopathy

As a microvascular complication of diabetes, DR affects the retinal blood vessels, thus leading to vision impairment. Dietary interventions that support glycemic control, reduce inflammation, and improve vascular health are crucial in managing and preventing DR. The MedDiet addresses all of these factors through its low glycemic load, high fiber content, and abundance of bioactive compounds [31,32].

Omega-3 fatty acids from fish can reduce retinal inflammation and vascular permeability, while antioxidants from fruits, vegetables, and extra virgin olive oil help neutralize oxidative stress. Polyphenols such as resveratrol (found in grapes and red wine) and hydroxytyrosol (from olive oil) exhibit neuroprotective and anti-angiogenic effects that may prevent the abnormal blood vessel growth seen in advanced DR. Observational studies and clinical trials have linked adherence to the MedDiet with a lower risk and the reduced progression of diabetic retinopathy in patients with type 2 diabetes [31,33].

Recent clinical research has investigated the potential role of omega-3 fatty acids in preventing or slowing the progression of DR. The most comprehensive study to date is the ASCEND-Eye trial, a sub-study of the larger ASCEND (A Study of Cardiovascular Events in Diabetes) randomized controlled trial [34]. It is worth noting that while omega-3 fatty acids have demonstrated anti-inflammatory and vascular benefits in other contexts, their efficacy in the prevention or treatment of diabetic retinopathy remains unproven in large-scale human trials. Further research may explore different dosages, formulations, or patient populations to fully elucidate any potential benefits.

## 4. Blue Light and Retinal Damage

### 4.1. Blue Light Exposure from Digital Screens and Artificial Lighting

Blue light is a high-energy, short-wavelength (400–500 nm) part of the visible light spectrum. It is naturally present in sunlight, but modern lifestyles have significantly increased our exposure to artificial sources of blue light. LED screens, smartphones, tablets, computers, etc., emit substantial levels of blue light. While exposure to natural blue light during the day helps regulate circadian rhythms and mood, prolonged and intense artificial exposure, especially at night, raises potential concerns about ocular health [35].

**Figure 1:**
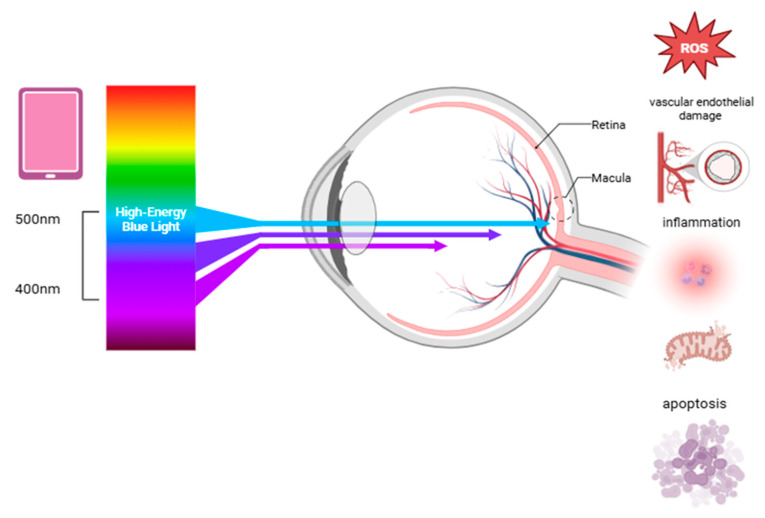
The effects of blue light on retinal health, highlighting the processes that contribute to retinal damage and may increase the risk of degenerative eye diseases. Abbreviation: ROS, reactive oxygen species.

High-energy short-wave blue light between 415 and 455 nm is the most harmful type, causing symptoms such as eye fatigue, dryness, blurred vision, and insomnia [10]. Unlike ultraviolet light, which is mostly absorbed by the cornea and the lens, blue light penetrates deeper and reaches the retina directly (Figure 1). This can cause cumulative photochemical stress on the retina and irreversible damage known as the “blue light hazard” [6]. Prolonged blue light exposure has been linked to eye diseases such as AMD, cataracts, and keratitis [10].

### 4.2. The Mechanisms of Oxidative Stress and Inflammation Caused by Blue Light

Blue light exposure can damage the eyes through several pathways, including oxidative stress, DNA damage, inflammation, mitochondrial dysfunction, apoptosis, impaired autophagy, and vascular endothelial damage (Figure 1) [36]. On the ocular surface, blue light can penetrate the eye and impact the corneal epithelial and endothelial cells, reducing their survival and increasing reactive oxygen species (ROS) and inflammatory markers like IL-1β [10]. The aging crystalline lens gradually absorbs more blue light, which can trigger ROS production in the lens epithelial cells, leading to apoptosis through the TGF-β/Smad3 pathway and contributing to cataract formation [6]. Damaged cells also release inflammatory signals, attracting immune cells and worsening tissue injury [9]. The retina is particularly vulnerable to oxidative stress due to its high metabolic activity and constant exposure to light and oxygen. Blue light can damage the retina primarily by inducing oxidative stress, especially in the retinal pigment epithelium (RPE) and photoreceptor cells. This damage is linked to the accumulation of lipofuscin, particularly its toxic component N-retinylidene-N-retinylethanolamin (A2E), which generates ROS under blue light [37,38]. These ROS are damaging because they react with cellular components like lipids, proteins, and the DNA. The retina contains large amounts of polyunsaturated fatty acids, particularly docosahexaenoic acid, which are especially prone to oxidation [33]. ROS cause endoplasmic reticulum stress, mitochondrial dysfunction, and lysosomal damage through the activation of pathways like MAPK and NF-κB [39,40], all contributing to RPE cell apoptosis. Blue light also disrupts calcium balance, further harming the mitochondria. Additionally, it elevates HIF-1α and VEGF expression, leading to retinal vascular endothelial dysfunction and potential neovascularization [41,42]. Prolonged exposure to low-intensity blue light can disrupt the retinal photoreceptors, while high-intensity blue light causes more severe damage. It inhibits cytochrome oxidase, disrupts mitochondrial function, reduces sodium–potassium ATPase activity, and leads to cell edema and photoreceptor degeneration [43]. Blue light also stimulates inflammatory signaling pathways, most notably NF-κB, a transcription factor that regulates genes involved in the immune response. The activation of NF-κB leads to the production of pro-inflammatory cytokines such as IL-6, TNF-α, and IL-1β. These cytokines create a chronic inflammatory environment that contributes to structural damage in the retina over time [44]. Rod and cone cells are particularly vulnerable due to rhodopsin and S-opsin, which enhance photon capture and increase light-induced damage. Short-wavelength LEDs exacerbate this by aggregating visual proteins and damaging the cone cells [45]. Moreover, blue light can compromise the blood–retinal barrier by damaging RPE tight junctions, allowing the infiltration of inflammatory cells into the retinal tissues. This perpetuates oxidative injury and accelerates retinal degeneration [46].

### 4.3. The Connection Between Blue Light and Retinal Conditions

AMD is a complex, progressive, multifactorial disease that develops gradually over the years. Several known risk factors, including age, smoking, nutritional deficiencies, sunlight exposure, and genetic predisposition, are involved in the pathogenesis of the disease [47]. Recently, it was shown that prolonged exposure to blue light could also contribute to the development and worsening of the clinical picture of AMD. Namely, accumulation of the retinoid A2E, a key component of lipofuscin, further increases the risk of retinal cell apoptosis with age, as A2E is particularly sensitive to high-energy blue light [38]. Although blue light exposure is not the sole cause of AMD, it is considered a contributing risk factor, especially in individuals with genetic predispositions or existing retinal vulnerabilities. Experimental studies using animal models and in vitro retinal cultures have shown that blue light accelerates RPE cell death, impairs photoreceptor renewal, and increases drusen-like deposits—hallmarks of early AMD [46,48,49]. Additionally, with age, the natural protective mechanisms against blue light decline. The crystalline lens gradually yellows, filtering more blue light, but this process is less effective in older adults or those who have undergone cataract surgery. The macular pigments lutein and zeaxanthin, which absorb blue light and neutralize ROS, also decrease with age and poor diets, further reducing the eye’s defenses [50,51].

A clinical study investigating the effects of blue light exposure on retinal health was conducted in 2021 by Li et al. [52]. This study combined a clinical pilot investigation with an animal model to assess the impact of low-intensity blue light, similar to that emitted by smartphones, on retinal function and structure. The clinical component consisted of 25 healthcare providers who were assigned into two groups based on their duration of daily screen use. Multifocal electroretinography (mf-ERG) was used to measure retinal function. The findings indicated that increased screen exposure was linked to lower mf-ERG amplitudes in macular regions, reflecting damaged retinal function [52].

In conclusion, as electronic device use increases, the incidence of blue-light-related eye damage is expected to rise, particularly among the young and the elderly. By 2050, the prevalence of eye diseases like myopia, diabetic retinopathy, glaucoma, and in particular AMD, as a leading cause of blindness in older adults, is predicted to grow significantly. Future research will likely focus on understanding the role of blue light in these conditions, especially in younger and older populations [53].

## 5. Mediterranean Herbs and Spices: Bioactive Compounds and Their Properties

Mediterranean herbs such as bay laurel, thyme, oregano, sage, and rosemary are rich in bioactive compounds with impressive health-promoting attributes, most significantly for the protection of retinal health. These herbs contain powerful phenolic compounds like rosmarinic acid, carnosic acid, and thymol, which have been extensively researched for their antioxidant, anti-inflammatory, and antimicrobial properties [54].

Rosmarinic acid, which is present in abundance in oregano and rosemary, has been shown to inhibit oxidative stress and reduce inflammatory responses in the retinal cells and may retard the progress of age-related macular degeneration [55]. Carnosic acid, which is predominantly present in rosemary and sage, offers neuroprotection through its ability to scavenge free radicals and modulate inflammatory signaling pathways [56]. Thymol, a major thyme constituent, possesses strong antimicrobial and anti-inflammatory activity that may contribute to ocular surface integrity and the prevention of retinal infections [57]. These findings emphasize the potential of Mediterranean spices as dietary sources of retinal-integrity- and -function-enhancing compounds. Regarding their antioxidant activity, they neutralize ROS, inhibit lipid peroxidation, and increase endogenous antioxidants like glutathione [35].

Their anti-inflammatory action involves the suppression of the NF-κB and COX-2 pathways, reductions in pro-inflammatory cytokines, and matrix metalloproteinase (MMP) blockade to protect the retinal structure. In retinal health, MMPs participate in retinal damage, as their activity has the capacity to degrade structural proteins and plays a role in inflammatory processes. MMP inhibition decreases tissue injury and inflammation, and since these are the same effects brought about through the inhibition of MMP activity (e.g., by rosmarinic acid), the compounds that inhibit MMP activity have the potential to protect the retinal tissues against damage [58,59].

Bioactive compounds that originate from these herbs can disrupt microbial membranes to prevent infections that can worsen retinal inflammation, in the context of antimicrobial action [60]. Rosmarinic acid, one of the prominent bioactive molecules in rosemary, sage, thyme, and oregano, possesses significant antioxidant, anti-inflammatory, and antimicrobial activities that may safeguard retinal function.

In Table 1, a summary of the key bioactive components found in the most commonly used aromatic herbs in the MedDiet are presented, together with the mechanisms through which they might support retinal health.

Studies indicate that combining different herbal compounds can produce synergistic effects beneficial for retinal health. Mixed natural antioxidants often target multiple pathways simultaneously, enhancing their overall efficacy compared to that of individual compounds alone [65]. Similarly, traditional herbal formulas containing multiple herbs exhibit additive or synergistic interactions among their phytochemicals, improving outcomes such as the inhibition of retinal neovascularization by downregulating pro-angiogenic factors. These synergistic effects arise because the combined compounds can scavenge ROS, suppress pro-inflammatory cytokines, inhibit MMPs, and modulate multiple signaling pathways involved in retinal degeneration [66]. Therefore, multi-compound herbal mixtures hold promise as more effective interventions for preventing or slowing retinal diseases than single isolated compounds.

The European Food Safety Authority (EFSA) evaluates the health claims on bioactive compounds in the MedDiet according to solid scientific evaluations based on three criteria: the characterization of a substance, definition of its claimed effect, and cause–effect relationships [67]. The EFSA authorizes health claims about the MedDiet’s components using robust evidence (e.g., olive oil polyphenols) while restricting generic or unsubstantiated claims. Nutrient profiling exemptions ensure that traditional foods like herbs and vegetable oils remain eligible. Most MedDiet staples are not novel foods, though novel processing methods may trigger regulatory review [68]. Regarding novel food regulations, they must balance innovation with respect for traditional diets. Whole herbs/spaces are excluded from novel food status, but standardized extracts or purified bioactive compounds (e.g., eugenol from cloves) may require authorization [69].

## 6. The Protective Effects of Mediterranean Herbs and Spices Against Blue-Light-Induced Retinal Damage

Alongside the broader emphasis on antioxidant-rich foods, the MedDiet may offer indirect protection against blue-light-induced retinal damage by way of the bioactive compounds found in herbs and spices. These protective effects are most likely achieved through systemic antioxidant and anti-inflammatory mechanisms [54,61,63,70]. Specific studies on Mediterranean herbs/spices for this purpose are limited, but the key components align with established certain protective pathways (an increase in antioxidant defense, anti-inflammatory effects, and anti-angiogenesis). While Mediterranean herbs and spices are likely to be beneficial in enhancing the systemic antioxidant defenses, their specific role in retinal protection from blue light still remains hypothetical, as there are no specific targeted studies. For now, the existing evidence supports their inclusion as part of a broader nutrient-dense diet for ocular health (Table 2).

Kim et al. investigated the therapeutic effects of eucalyptol on diabetic retinal microvascular defects [71]. High glucose levels in diabetic conditions increase Aβ production, which damages the retinal cells. Eucalyptol significantly reduced the Aβ levels in both human retinal microvascular endothelial (RVE) cells and diabetic mice. Eucalyptol blocked apoptosis (cell death) in the retinal cells by restoring protective proteins (bcl-2) and reducing harmful ones (bax and caspase-12). Eucalyptol also reduced endoplasmic reticulum stress, which is a key contributor to retinal damage in diabetes. It blocked the PERK-eIF2α-ATF4-CHOP signaling pathway, which is activated by Aβ and high glucose. Eucalyptol significantly reduced the Aβ levels in both human RVE cells and diabetic mice (Table 2).

Another study on the effects of rosmarinic acid on retinal neovascularization showed its anti-angiogenic properties, as it suppressed retinal neovascularization by causing cell cycle arrest in the G2/M phase and increasing the expression of p21WAF1, a protein that regulates cell growth. It inhibited the proliferation and tube formation of the retinal endothelial cells in a dose-dependent manner. In a mouse model of retinopathy of prematurity, rosmarinic acid effectively reduced retinal neovascularization without causing retinal toxicity (Table 2) [72].

In animals exposed to intense light, carnosic acid administration preserved photoreceptor function and morphology. This protection is linked to the activation of the Nrf2 pathway, leading to the upregulation of endogenous antioxidant enzymes and a reduction in oxidative damage markers such as hyperoxidized peroxiredoxin 2 (Prx2).

Carnosic acid treatment has been observed to decrease the production of pro-inflammatory cytokines, including IL-1β, IL-6, and TNF-α, thereby reducing inflammation-mediated retinal damage [76]. In the Pde6b (rd10) mouse model of retinitis pigmentosa, carnosic acid treatment resulted in the preservation of the photoreceptor cells and retinal structure. This neuroprotective effect is associated with the inhibition of the oxidative and endoplasmic reticulum stress pathways [77].

Inflammation has a significant role in the progression of retinal degenerative diseases. Eugenol has been shown to suppress the production of pro-inflammatory cytokines, such as IL-1β, IL-6, and TNF-α, and may contribute to reducing retinal inflammation and subsequent neuronal damage [78].

Thymol’s antioxidant and anti-inflammatory effects observed in neuronal models suggest its potential therapeutic value in retinal neurodegenerative conditions [79]. Oxidative stress and inflammation are key contributors to retinal degenerative diseases such as AMD and retinitis pigmentosa; thus, thymol’s properties could be beneficial in this context. In a study involving a rotenone-induced model of Parkinson’s disease in rats, thymol treatment significantly reduced dopaminergic neuronal loss, oxidative stress markers, and pro-inflammatory cytokines such as IL-1β, IL-6, and TNF-α. Additionally, thymol enhanced the activity of antioxidant enzymes like superoxide dismutase (SOD) and catalase (CAT) and increased glutathione (GSH) levels, indicating a bolstered endogenous antioxidant defense system [80].

## 7. Future Directions: Dietary Recommendations for Eye Health

Dietary recommendations for eye health should emphasize the integration of Mediterranean spices and herbs—such as bay laurel, thyme, oregano, sage, and rosemary—into daily meals given their rich content of bioactive compounds like quercetin, luteolin, and rosmarinic acid, which possess strong antioxidant and anti-inflammatory properties [70]. These phytochemicals may help combat oxidative stress and inflammation, both key contributors to retinal degeneration [81]. The development of targeted nutritional interventions, such as supplements or functional foods enriched with these specific compounds, holds promise for preventing or slowing the progression of retinal diseases [81]. Incorporating Mediterranean herbs such as bay laurel, thyme, oregano, sage, and rosemary into daily diets offers promising avenues for protecting eye health.

Rosemary contains potent antioxidants, including rosmarinic and carnosic acids, which have demonstrated protective effects against oxidative stress in the eyes. Studies suggest that rosemary extract may slow the progression and severity of AMD and delay the onset of cataracts, though most of this research has used concentrated extracts rather than culinary amounts. Incorporating rosemary into meals—such as with roasted vegetables or in soups or teas—may offer some benefit as part of an overall antioxidant-rich diet [82].

Both thyme and sage are rich in lutein, a carotenoid crucial for the health of the macula. Lutein acts as a natural shield, protecting the retina from oxidative damage and blue light exposure [83].

Oregano is another Mediterranean herb high in antioxidants and anti-inflammatory compounds. While specific clinical studies on oregano and eye health are limited, its general antioxidant properties support a reduction in oxidative stress, a key factor in the development of AMD and cataracts [84].

While direct evidence for bay laurel’s impact on eye health is limited, it is a staple in Mediterranean cuisine and contributes to the overall antioxidant profile of this diet.

In addition to the positive retinal health benefits of herbs and spices, the MedDiet is characterized by a high intake of fruits, vegetables, whole grains, legumes, nuts, olive oil, and herbs and is associated with a significantly reduced risk of age-related macular degeneration and other ocular diseases. Key nutrients for eye health found abundantly in this diet include lutein and zeaxanthin (leafy greens, herbs), vitamins C and E (citrus, nuts, and olive oil), omega-3 fatty acids (seafood and nuts), and zinc (legumes, seeds, and seafood) [29].

One suggestion is to incorporate Mediterranean herbs into daily meals to enhance antioxidant and anti-inflammatory intake and then combine herbs with other Mediterranean staples—leafy greens, colorful vegetables, nuts, olive oil, and fish—for synergistic effects. All of this alongside a reduction in salt, using herbs and spices to flavor foods in place of salt, can support both eye and cardiovascular health [85].

To advance clinical applications, further research is needed to isolate, characterize, and test the efficacy of these bioactive molecules in human trials, as well as to explore their synergistic effects with other MedDiet components like healthy fats and carotenoids [70]. Such studies could pave the way for precision nutrition strategies tailored to individuals at risk of blue-light-induced retinal degeneration and other ocular conditions. Suggestions for further research into bioactive compounds for clinical applications should prioritize human clinical trials to validate the preclinical findings, particularly for Mediterranean spices. Studies should investigate the synergistic interactions between carotenoids (e.g., lutein, zeaxanthin) and polyphenols (e.g., anthocyanins, quercetin) to optimize the antioxidant and anti-inflammatory effects while addressing potential absorption conflicts (e.g., the inhibition of lutein uptake by certain polyphenols) [86]. The research must also focus on bioavailability enhancements through novel delivery systems (e.g., nanoencapsulation) to improve the penetration of the ocular tissue by compounds like resveratrol [66,86]. Additionally, long-term trials are needed to assess the efficacy of bioactive combinations in preventing AMD and diabetic retinopathy, with an emphasis on the dose–response relationships and safety profiles [87]. Mechanistic studies should explore molecular pathways, including VEGF and TNF-α inhibition, to identify targets for precision nutrition strategies [88]. Finally, standardized extraction methods for spice-derived compounds and their integration into functional foods warrant exploration to bridge dietary recommendations and therapeutic applications [89].

## 8. Conclusions

The MedDiet, rich in plant-based foods, healthy fats, and herbs, has been associated with a variety of health benefits, including cardiovascular, neuroprotective, and anti-inflammatory effects. Recent research suggests that certain components of the MedDiet, particularly herbs such as bay leaf, thyme, oregano, sage, and rosemary, may play a key role in protecting the retina from oxidative damage, a key factor in blue-light-induced retinal degeneration. At the moment, there is a scarcity of evidence directly supporting the potential of these MedDiet staples; however, the findings presented in this review highlight the promising role of Mediterranean herbs and spices—as well as the bioactive compounds found in them—in preventing retinal damage, as well as other eye conditions related to modern digital lifestyles and the heavy use of blue-light-emitting devices.

## Figures and Tables

**Table 1:** Selected aromatic herbs, their key bioactive compounds, and their mechanisms related to retinal health.

Aromatic Herbs	Key Bioactive Compounds	Mechanisms Supporting Retinal Health	Clinical Implications
Rosemary (*Rosmarinus officinalis*)	Rosmarinic acid Carnosic acid	Antioxidant (scavenges ROS, upregulates glutathione), anti-inflammatory (inhibits NF-κB, COX-2), antimicrobial [61]; RA inhibits aldose reductase, an enzyme linked to diabetic retinopathy; CA combats oxidative stress in retinal degeneration [54].	RA’s inhibition of aldose reductase may help manage diabetic retinopathy; antioxidant/anti-inflammatory effects may slow AMD progression.
Sage (*Salvia* spp.)	Rosmarinic acid Carnosic acid	Antioxidant (reduces lipid peroxidation), enhances antioxidant defenses [61].	Potential for retinal protection via antioxidant and anti-inflammatory effects.
Thyme (*Thymus vulgaris*)	Thymol Rosmarinic acid	Antioxidant, anti-inflammatory (suppresses cytokines, inhibits MMPs), antimicrobial [54].	Potential protection against retinal inflammation and infection.
Oregano (*Origanum vulgare*)	Thymol Rosmarinic acid	Antioxidant, anti-inflammatory (suppresses cytokines, inhibits MMPs), antimicrobial [54].	Potential protection against retinal inflammation and infection.
Bay Laurel (*Laurus nobilis*)	Rosmarinic acid, ferulic acid, caffeic acid [62]	Antioxidant [63,64].	Possible antioxidant support of retinal health [63].

Abbreviations: AMD, age-related macular degeneration; CA, carnosic acid; COX-2, cyclooxygenase-2; MMPs, matrix metalloproteinases; NF-κB, nuclear factor kappa B; RA, rosmarinic acid; ROS, reactive oxygen species.

**Table 2:** Ocular-health-protective effects of natural bioactive compounds found in Mediterranean herbs and spices in experimental models.

Bioactive Compound	Source	Experimental Model and Treatments	Observations	Reference
Eucalyptol (1,8-cineole)	Monoterpene oxide primarily sourced from *Eucalyptus* species (e.g., *Eucalyptus globulus*); also found in *Laurus nobilis* (bay laurel), *Rosmarinus officinalis* (rosemary), *Salvia officinalis* (sage), *Cinnamomum camphora* (camphor tree), and *Ocimum basilicum* (basil).	33 mM glucose-exposed human RVE cells and diabetic db/db mice. In vitro: 1–20 μM of eucalyptol, 3-day pretreatment In vivo: 10 mg/kg of oral eucalyptol, 8 weeks of treatment	Reduced Aβ protein production in glucose-treated RVE cells and diabetic mouse eyes and mitigated apoptosis in Aβ-exposed RVE cells and diabetic retinal cells. Blocked Aβ-mediated ER stress by inhibiting the PERK-eIF2α-ATF4-CHOP pathway. Activated the Ang-1/Tie-2 signaling pathway and inhibited the Ang-2 and VEGF expression in diabetic models. Reversed the Aβ-induced suppression of the junction proteins VE-cadherin and occludin-1 in the RVE cells and reduced Aβ-induced permeability in the RVE cells. Oral eucalyptol diminished vascular leakage in diabetic retinal vessels.	[71]
Rosmarinic acid (RA)	A polyphenolic compound naturally occurring mainly in plants from the *Lamiaceae* family, especially in rosemary (*Salvia rosmarinus*/*Rosmarinus officinalis)*, as well as in *Melissa officinalis* (lemon balm), *Perilla frutescens* (perilla), *Ocimum basilicum* (basil), *Thymus vulgaris* (thyme), and *Origanum vulgare* (oregano).	Retinal endothelial cells and an in vivo mouse model of oxygen-induced retinopathy of prematurity. In vitro: 10–100 μM of RA, 24 h In vivo: 50 µM of RA IVT injected on postnatal day P14	Inhibited retinal neovascularization, which is related to cell cycle arrest with an increase in p21^WAF1^. Antiproliferative activity in the retinal endothelial cells was related to G2/M phase cell cycle arrest in a dose-dependent manner.	[72]
Carnosic acid (CA)	A phenolic diterpene primarily found in plants from the *Lamiaceae* family, especially in rosemary (*Rosmarinus officinalis*) and common sage (*Salvia officinalis*); also present in *Salvia lavandulifolia* and other *Salvia* species.	H_2_O_2_-induced toxicity in retina-derived cell lines (human ARPE-19 and mouse photoreceptor-derived 661W cell lines) and light-induced stress in 5–6-week-old Sprague-Dawley rats. In vitro: 10 μM of CA for 24 h In vivo: 25 mg/kg/day through IP injection for 6 days	Induced antioxidant phase 2 enzymes and reduced the formation of hyperoxidized peroxiredoxin (Prx2). Protected the retinas in vivo from light-induced damage, producing significant improvements in the thickness of the outer nuclear layer and electroretinogram activity.	[73]
Eugenol	A phenylpropene derivative found predominantly in clove oil (from *Syzygium aromaticum)*; also present in *Cinnamomum verum* (true cinnamon), *Ocimum sanctum* (holy basil/tulsi), *Pimenta dioica* (allspice), and *Laurus nobilis* (bay leaf).	An acute corneal pain model in rats. Systemic: 50, 100, and 200 mg/kg clove oil, SC Topical: 25, 50, and 200 µg clove oil/eye, 25, 50, and 100 µg eugenol/eye	Had analgesic and local anesthetic effects on the cornea, with potential applications in managing ocular discomfort, through the opioidergic and cholinergic systems.	[74]
Thymol	A monoterpenoid phenol primarily found in *Thymus vulgaris* (thyme) and *Origanum vulgare* (oregano); also present in *Monarda didyma* (bee balm), *Satureja montana* (winter savory), and *Ajwain* (*Trachyspermum ammi*).	Isolated goat lenses for modeling of cataracts induced by high-glucose exposure. In vitro: 20 to 60 µg/mL, 3 days	Prevented cataract formation through the inhibition of aldose reductase and a reduction in oxidative stress (a decrease in lipid peroxidation levels).	[75]

Abbreviations: Aβ, amyloid-β; ARPE-19, adult retinal pigment epithelium; ATF4, activating transcription factor 4; CHOP, C/EBP homologous protein; eIF2α, eukaryotic initiation factor 2 alpha subunit; ER, endoplasmic reticulum; IVT, intravitreally; PERK, protein kinase R (PKR)-like ER kinase; retinal pigment epithelium; RVE, retinal microvascular endothelial; SC, subcutaneously; VEGF, vascular endothelial growth factor.

## Abbreviations

The following abbreviations are used in this manuscript:

AMD	Age-related macular degeneration
ASCEND	A Study of Cardiovascular Events in Diabetes
A2E	N-retinylidene-N-retinylethanolamin
BDNF	Brain-derived neurotrophic factor
CA	Carnosic acid
CRP	C-reactive protein
DES	Digital eye strain
DNA	Deoxyribonucleic acid
DR	Diabetic retinopathy
EFSA	European Food Safety Authority
ER	Endoplasmic reticulum
HDL	High-density lipoprotein
HIF-1α	Hypoxia-inducible factor 1-alpha
ICAM-1	Intercellular adhesion molecule-1
IL-1β	Interleukin-1 beta
IL-6	Interleukin-6
IL-10	Interleukin-10
LDL	Low-density lipoprotein
LED	Light-emitting diode
MAPK	Mitogen-activated protein kinase
MedDiet	Mediterranean diet
mf-ERG	Multifocal electroretinography
MMP	Matrix metalloproteinase
NF-κB	Nuclear factor-kappa B
PAF	Platelet-activating factor
PREDIMED	Prevention with Mediterranean Diet
RA	Rosmarinic acid
ROS	Reactive oxygen species
RPE	Retinal pigment epithelium
RVE	Retinal microvascular endothelial
TGF-β	Transforming growth factor beta
TNF-α	Tumor necrosis factor-alpha
UNESCO	United Nations Educational, Scientific and Cultural Organization
VCAM-1	Vascular cell adhesion molecule 1
VEGF	Vascular endothelial growth factor
